# Metabolomic profiles of an atherogenic TMAO-dietary pattern among postmenopausal women

**DOI:** 10.1007/s00394-025-03792-w

**Published:** 2025-09-04

**Authors:** Kaelyn F. Burns, Rachael Hageman Blair, Michael J. LaMonte, Jean Wactawski-Wende, Kathryn M. Rexrode, Raji Balasubramanian, Fred K. Tabung, Linda Snetselaar, Amy E. Millen

**Affiliations:** 1 Department of Epidemiology and Environmental Health, University at Buffalo, 270 Farber Hall, Buffalo, NY 14214 USA; 2 Department of Biostatistics, University at Buffalo, Buffalo, NY 14214 USA; 3 Division of Women’s Health, Department of Medicine, Brigham and Women’s Hospital, Boston, MA 02115 USA; 4 Department of Biostatistics and Epidemiology, University of Massachusetts Amherst, Amherst, MA 01003 USA; 5 Department of Internal Medicine, The Ohio State University, Columbus, OH 43210 USA; 6 Department of Epidemiology, University of Iowa, Iowa, IA 52242 USA

**Keywords:** Postmenopausal women, Atherogenic diet, Choline, Trimethylamine *n*-oxide, Metabolism, Metabolomics

## Abstract

**Purpose:**

We previously identified a dietary pattern (DP) associated with plasma trimethylamine *n*-oxide (TMAO) and choline, the TMAO-DP, where higher scores represent more atherogenic potential of the diet. The mechanisms linking dietary intake to the presence of choline and TMAO in the plasma, and by which TMAO may influence atherosclerosis in humans require further clarification. The objective was to evaluate associations between the TMAO-DP and metabolomic profiles in postmenopausal women from the Women’s Health Initiative (WHI).

**Methods:**

This cross-sectional analysis used baseline WHI data. Dietary intake was assessed using a modified Block food frequency questionnaire. Liquid chromatography-mass spectrometry was used to measure 446 metabolites from plasma samples. Linear regression models were used to evaluate associations between the TMAO-DP and metabolites. Metabolites associated with the TMAO-DP were first identified in a Discovery Sample (WHI Hormone Trials participants; n = 1117) and then were replicated in an independent sample, the Replication Sample (WHI Observational Study participants; n = 870). Pathway enrichment analysis was used to identify overrepresented metabolic pathways in our set of significant metabolites.

**Results:**

There were 132 metabolites associated with the TMAO-DP in the Discovery Sample, and 83 of those were replicated. In the Replication Sample, metabolite classes positively associated with the TMAO-DP included phosphatidylcholine plasmalogen, phosphatidylethanolamine plasmalogen, and acyl carnitine, and the metabolite classes negatively associated included phosphatidylcholine and cholesterol ester. Overrepresented pathways included pathways of lipid metabolism.

**Conclusion:**

This study provides insights into the metabolic processes linking dietary intake to TMAO production, and TMAO to atherosclerosis, among postmenopausal women.

**Supplementary Information:**

The online version contains supplementary material available at 10.1007/s00394-025-03792-w.

## Introduction

Diet is a major risk factor for atherosclerotic cardiovascular disease (CVD) [1]; however, not all mechanisms involved have been completely elucidated. One mechanism of interest regarding how diet may influence CVD involves the production of trimethylamine N-oxide (TMAO), a proposed proatherosclerotic metabolite [2, 3]. The gut microbiome can metabolize dietary choline to produce trimethylamine (TMA), which can then be transported to the liver and oxidized to produce TMAO [4]. Experimental studies have demonstrated that consuming foods that are rich sources of choline (e.g., red meat, eggs, fish) increases circulating TMAO [5–8]. However, dietary fiber can suppress the conversion of choline to TMA because fiber suppresses the synthesis of intermediates (i.e., glycine) used by the microbiome in this metabolic reaction [9–11]. Therefore, it is important to understand how overall dietary intake leads to TMAO concentrations, potentially leading to the progression of atherosclerosis. We previously developed the TMAO Dietary Pattern (TMAO-DP), which is high in food sources of choline and low in food sources of fiber; and we validated that higher compared to lower TMAO-DP scores are associated with higher concentrations of plasma TMAO and choline [12].

TMAO has been shown to induce and progress atherosclerosis in mouse models through multiple mechanisms. These include upregulating scavenger receptors on macrophages thereby increasing uptake of cholesterol and foam cell formation [2], reducing reverse cholesterol transport [3], and activating inflammatory pathways in the vasculature [13]. In plasma isolated from healthy human study participants, TMAO increased platelet adhesion by promoting the release of intracellular calcium stores [14]. Many epidemiologic studies have demonstrated positive associations between TMAO and CVD events [3, 15–19], although not all studies have found consistent results [20, 21]. Altogether, the mechanisms by which overall dietary intake leads to TMAO and how TMAO may lead to atherosclerosis in humans requires further clarification. Metabolomics can help us better understand the underlying metabolic processes and pathways linking diet to disease outcomes [22, 23].

The TMAO-DP was developed using data from participants in the Women’s Health Initiative (WHI) [12]. The purpose of the current study was to evaluate the association between the TMAO-DP and metabolomic profiles among a separate set of participants from the WHI. The aims of this analysis were to further the understanding of the underlying metabolic processes that occur in the metabolism of foods that contributes to the presence of choline and TMAO in the plasma, and provide insights into the mechanisms by which TMAO may induce or progress atherosclerosis in humans. Postmenopausal women are a high-risk group for developing atherosclerotic CVD [1], and age-related hormonal changes can impact metabolic processes [24], making this an epidemiologic and clinically relevant population to investigate this diet-metabolomic association in.

## Methods

### Study sample

The WHI Observational Study (OS) and Hormone Trials (HTs) are multi-center studies of postmenopausal women, aged 50–79 years at enrollment (1993–1998) [25]. The WHI OS is a prospective cohort study designed to explore predictors and incidence of morbidity and mortality in aging women (n = 93,676). The WHI HTs were randomized, placebo-controlled trials designed to understand if postmenopausal hormone therapy was associated with risk of heart disease, major osteoporotic fractures, and cancer (n = 27,347). The Metabolomics of Coronary Heart Disease in the WHI study was a nested case–control study within WHI OS and HTs which aimed to examine metabolites associated with incident coronary heart disease (CHD) and examine how hormone therapy impacts metabolomic profiles in participants [26]. At WHI OS or HTs entry, all participants were free from CHD. To be included in the nested case–control study, participants had to have at least 250 µL of plasma stored from the baseline assessment in the WHI OS or HTs [27]. Cases of CHD with sufficient plasma stored were identified. In the WHI HTs, controls were frequency matched to cases on 5-year age intervals, race/ethnicity, 2-year enrollment time intervals, prior CVD conditions, and HT trial arm. In the OS sample, any participants who reported at baseline that they had experienced a prior CVD event were excluded. Controls from the OS were frequency matched to cases on 5-year age intervals, race/ethnicity, 2-year enrollment year intervals, and hysterectomy status. Further details on the selection procedures used in this ancillary study have been previously described [27, 28].

**Fig. 1:**
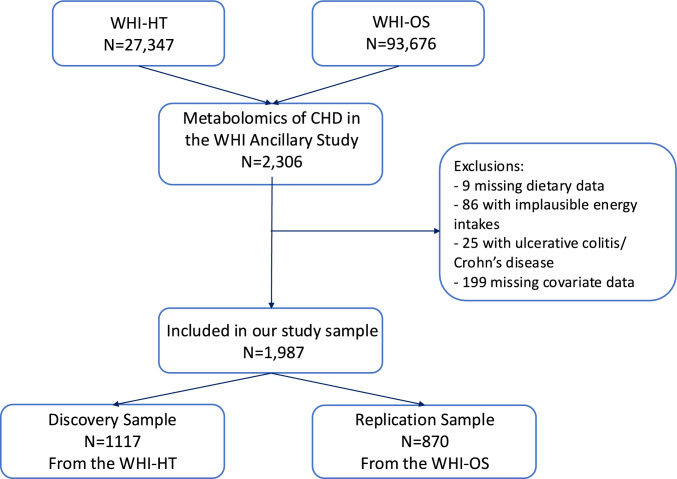
Study sample flow chart. CHD, coronary heart disease; HT, Hormone Trials; OS, Observational Study; WHI, Women’s Health Initiative

This cross-sectional analysis used baseline data from the Metabolomics of Coronary Heart Disease in the WHI study (n = 2306) [28]. Of those, 9 were missing dietary data and 86 reported implausible energy intakes (≤ 600 kcal/d or ≥ 5000 kcal/day) and were excluded from this analysis. We also excluded 25 participants who reported having ulcerative colitis or Crohn’s disease at baseline because these diseases may influence both dietary intake and metabolism. The sample was further restricted to include only those who had complete data on covariates (4 were missing body mass index [BMI], 72 were missing smoking packyears, 127 were missing recreational physical activity). The total number of participants excluded for missing covariate data was 199. This resulted in a sample size of 1987. There were 1117 women from the WHI HT, and 870 from the WHI OS, herein referred to as the Discovery and Replication samples, respectively (Fig. 1). This method of analyzing the OS and HT women separately aligns with recommendations to identify a Replication Sample that is similar, but independent from your Discovery Sample [29]. In addition, assigning the HT women as the Discovery Sample and the OS women as the Replication Sample has been done in previous publications using this metabolomics data [30, 31].

The WHI project was reviewed and approved by the Fred Hutchinson Cancer Research Center (Fred Hutch) IRB in accordance with the U.S. Department of Health and Human Services regulations at 45 CRF 46 (approval number: IR# 3467-EXT). Participants provided written informed consent to participate. The WHI Clinical Trial Registration number is NCT00000611.The Metabolomics of Coronary Heart Disease in the WHI study was approved by Partners Human Research Committee, the Institutional Review Board of Brigham and Women’s Hospital.

### Dietary assessment and TMAO dietary pattern (TMAO-DP) score

Dietary data was collected using a modified Block Food Frequency Questionnaire (FFQ) with 122-line items [32]. The FFQ was administered at WHI OS and HTs baseline and was used to examine participants’ habitual dietary intake over the previous three months. FFQ line items were converted into MyPyramid food groups as described in detail in the MyPyramid Equivalents Database (MPED) [33].

The TMAO-DP was developed in an ancillary study of WHI OS (n = 1724; mean age 66.6 years) to represent the atherogenic potential of the diet specifically as it relates to the choline → TMAO metabolic pathway [12]. Briefly, a reduced rank regression model was conducted with 27 energy-adjusted and log-transformed MPED food groups in serving equivalents (collectively referred to as “food groups” moving forward) as the exposure variables and log-transformed plasma concentrations of choline (μmol/L) and TMAO (μmol/L) as the outcome variables. The model resulted in a linear combination of those food groups that is predictive of plasma TMAO and choline. The top factor loadings from the RRR model were retained, and the TMAO-DP score is computed from the sum of the MPED food groups weighted by the factor loading variables. The food groups with the highest positive factor loadings are discretionary solid fat, discretionary oil, white potatoes, processed meat (franks, sausages, luncheon meats), unprocessed red meat (beef pork, veal, lamb, and game), and eggs. The food groups with the lowest negative factor loadings are yogurt, fruits, added sugar, and starchy vegetables.

Further details on the food groups in the TMAO-DP, and on the methods of development, can be found in the dietary pattern development paper [12] and on the MPED [33]. The dietary pattern development method utilized here results in a dietary pattern score with a mean of zero in the study sample. The TMAO-DP score was standardized to have a standard deviation of one prior to analyses. Higher TMAO-DP scores represent higher atherogenic potential of the diet as the score correlates positively with circulating choline and TMAO concentrations.

### Metabolite assessment

Metabolite data used in this study have been deposited in and are available from the dbGaP database under dbGaP accession phs001334.v1.p3.

Plasma samples were collected using EDTA tubes at the WHI OS and HT baseline following an overnight fast (≥ 8 h) and were stored at -80 degrees Celsius until processing. Metabolomics profile measurements were conducted at the Broad Institute (Boston, MA) using four different liquid chromatography-mass spectrometry (LC–MS) methods depending on the type of metabolites being measured. The identities of all the metabolites included in this analysis were confirmed using authentic reference standards or reference samples. Details of the LC–MS methods have been described previously [28]. In short, water soluble metabolites in positive ionization mode (hydrophilic interaction chromatography (HILIC)-pos) were analyzed using an LC–MS system made of a Shimadzu Nexera X2 U-HPLC (Shimadzu Corp.; Marlborough, MA) and a Q Exactive hybrid quadrupole orbitrap mass spectrometer (Thermo Fisher Scientific; Waltham, MA). Water soluble metabolites in negative ionization mode (HILIC-neg) were analyzed using an LC–MS system made of an AQUITY UPLC system (Waters; Milford, MA) and a 5500 QTRAP mass spectrometer (SCIEX; Framingham, MA). Polar and non-polar lipids (C8-pos) were analyzed using an LC–MS system made up of a Shimadzu Nexera X2 U-HPLC (Shimadzu Corp.; Marlborough, MA) and an Exactive Plus orbitrap mass spectrometer (Thermo Fisher Scientific; Waltham, MA). Free fatty acids and bile acids (C18-neg) were analyzed using an LC–MS system made up of a Shimadzu Nexera X2 U-HPLC (Shimadzu Corp.; Marlborough, MA) and a Q Exactive hybrid quadrupole orbitrap mass spectrometer (Thermo Fisher Scientific; Waltham, MA).

For each of the four LC–MS methods, pooled plasma samples were included after every 20 samples as a method of quality control. To minimize the influence pre-analytic factors (e.g., sample collection, aliquoting, transporting, storage), metabolites should be standardized before analysis [34]. Metabolites were standardized by calculating the ratio of the value of the sample to the nearest pooled reference sample, and multiplying by the median of all reference samples, as previously described [28].

In total, 509 metabolites were identified. Coefficients of variation (CV) were calculated using pooled plasma samples of 800 participants from the WHI OS. Metabolites with median CVs > 20% were excluded from analysis, as has been done previously in this data set [30, 31]. This removed 38 metabolites. In addition, metabolites with > 20% missing values were excluded [35]. This removed an additional 25 metabolites, resulting in 446 metabolites remaining for analysis. When metabolites were below the limit of detection, half of the lowest observed value was imputed [28].

Metabolites were classified by their chemical taxonomy direct parent (referred to as the metabolite category) by using their Human Metabolome Database (HMDB) identification number (IDs) (Supplemental Table 1) [36]. HMDB IDs were then matched to Kyoto Encyclopedia of Genes and Genomes (KEGG) [37] IDs using the MetaboAnalyst Metabolite ID Conversion tool [38]. Lipid metabolites are reported at the species level using sum composition annotations (i.e., number of carbon atoms: number of double bond equivalents) [39].

**Table 1:** Participant demographic and health behavior characteristics in the Discovery Sample and the Replication Sample, by quartiles of the TMAO-DP

	WHI-HT Discovery Sample N = 1117			WHI-OS Replication Sample N = 870		
	Q1 n = 279	Q4 n = 279	*p*-value	Q1 n = 217	Q4 n = 217	p-value
Age (years), mean (SD)	67.6 (6.4)	65.4 (7.1)	**0.0012**	68.1 (6.6)	66.3 (7.2)	**0.0146**
Race*, n (%)			**0.0335**			**0.0014**
AI/AN	1 (0.4)	0		0	2 (0.9)	
Asian	0	3 (1.1)		6 (2.8)	4 (1.8)	
Native hawaiian/Other PI	0	1 (0.4)		0	1 (0.5)	
Black	30 (10.8)	41 (14.7)		19 (8.8)	47 (21.7)	
White	243 (87.1)	224 (80.3)		186 (85.7)	152 (70.1)	
More than one race	3 (1.1)	4 (1.4)		2 (0.9)	2 (0.9)	
Unknown/Not reported	2 (0.7)	6 (2.2)		4 (1.8)	9 (4.2)	
Ethnicity, n (%)			**0.0034**			0.6119
Not Hispanic/Latino	274 (98.2)	259 (93.8)		205 (94.5)	205 (94.5)	
Hispanic/Latino	5 (1.8)	17 (6.1)		10 (4.6)	6 (2.8)	
Unknown/Not reported	0	3 (1.1)		2 (0.9)	6 (2.8)	
Body mass index (kg/m^2^), mean (SD)	28.5 (5.2)	30.4 (6.6)	**0.0007**	26.6 (5.3)	29.3 (6.5)	**0.0001**
Education level, n (%)			** < 0.0001**			** < 0.0001**
Some high school or less	14 (5.1)	28 (10.1)		5 (2.4)	24 (11.1)	
High school	36 (13.0)	95 (34.4)		28 (13.2)	55 (25.5)	
Vocational school	119 (43.1)	112 (40.6)		79 (37.1)	91 (42.1)	
College	28 (10.1)	16 (5.8)		29 (13.6)	14 (6.5)	
Graduate degree	79 (28.6)	25 (9.1)		72 (33.8)	32 (14.8)	
Smoking status, n (%)			**0.0008**			**0.0022**
Never smoked	139 (49.8)	134 (48.0)		117 (53.9)	107 (49.3)	
Past smoker	114 (40.9)	97 (34.8)		89 (41.0)	79 (36.4)	
Current smoker	26 (9.3)	48 (17.2)		11 (5.1)	31 (14.3)	
Pack years of smoking, mean (SD)	10.9 (19.2)	15.9 (24.4)	**0.0240**	0.9 (1.2)	1.2 (1.3)	0.1186
Recreational physical activity (MET-hrs/week), mean (SD)	13.3 (14.3)	6.9 (9.9)	** < 0.0001**	16.1 (14.0)	8.3 (11.4)	** < 0.0001**
Hormone replacement therapy, n (%)			–			0.1829
Never used or Past user	279 (100.0)	279 (100.0)		131 (60.4)	145 (66.8)	
Current user	0	0		86 (39.6)	72 (33.2)	
Taking hypertension medication, n (%)			0.1675			0.1655
No	238 (85.3)	243 (87.1)		184 (84.8)	183 (84.3)	
Yes	41 (14.7)	36 (12.9)		33 (15.2)	34 (15.7)	
Taking anti-diabetic medication, n (%)			0.0591			**0.0017**
No	253 (90.7)	245 (87.8)		212 (97.7)	195 (89.9)	
Yes	26 (9.3)	34 (12.2)		5 (2.3)	22 (10.1)	
Taking cholesterol medication, n (%)			0.5357			0.3895
No	234 (83.9)	242 (86.7)		188 (86.6)	198 (91.2)	
Yes	45 (16.1)	37 (13.3)		29 (13.4)	19 (8.8)	
History of myocardial infarction, n (%)			0.6100			–
No	248 (88.9)	252 (90.3)		217 (100.0)	217 (100.0)	
Yes	31 (11.1)	27 (9.7)		0	0	
History of stroke, n (%)			0.0624			–
No	270 (96.8)	268 (96.1)		217 (100.0)	217 (100.0)	
Yes	9 (3.2)	11 (3.9)		0	0	

Race variable was collapsed to White versus Non-White categories for the chi-squared test. Ethnicity variable was collapsed to Hispanic/Latino versus Non-Hispanic/Latino for the chi-squared test. From the WHI-HT, 6 participants are missing data on education. From the WHI-OS, 10 participants are missing data on education

Boldface indicates statistical significance at a p-value<0.05

TMAO-DP, trimethylamine N-oxide dietary pattern; WHI, Women’s Health Initiative; HT, Hormone Trials; OS, Observational Study; Q, quartile; SD, standard deviation; AI/AN, American Indian/Alaska Native; MET, metabolic equivalents

### Demographic and health characteristic data

Demographic data on age, race, ethnicity, education, and health behavior data on smoking packyears, recreational physical activity (metabolic equivalent hours per week (MET-hrs/week)), CVD history, personal hormone replacement therapy use, and medication use were collected at WHI baseline via questionnaire [40, 41]. Height and weight were measured in a clinical setting by study staff. For those who were in the HTs, trial arms assigned for the HTs as well as the WHI dietary modification trial and calcium and vitamin D trial (if applicable) are also reported.

### Statistical analysis

#### Sample characteristics and dietary intake

The TMAO-DP was applied to the WHI HT sample (Discovery Sample) and the WHI OS sample (Replication Sample), which resulted in a single value (score) per participant to estimate adherence to the dietary pattern (e.g., a higher score reflects greater adherence). Participant demographic and health behavior characteristics by quartiles of the TMAO-DP score were examined separately for the Discovery Sample and the Replication Sample using one-way analysis of variance (ANOVA) and chi-squared tests. Mean food group intake and macronutrient and micronutrient intake were also evaluated by quartiles of the TMAO-DP, separately for the Discovery Sample and the Replication Sample using ANOVA. Food groups, macronutrients, and micronutrients were energy adjusted using the density methods, and presented as intake per 1000 kcals [42].

#### Associations between the TMAO-DP and metabolites

The distributions of metabolites were visualized using histograms. To address skewness, all metabolites were log-transformed before conducting analyses. Principal components were used for anomaly detection and to examine any multivariate differences that may be observed between the Discovery and Replication samples. Principal component (PC) 1 and PC 2 explained the most variation, at 18.2% and 14.8% respectively. Score plots of PC 1 through PC 5, explaining 47.8% of the variation, did not reveal any significant outliers and all samples were retained for analyses. Furthermore, no significant separation was observed across different covariates in the PC score plots (Supplemental Fig. 1).

In the Discovery Sample, we examined associations between the TMAO-DP and metabolomic profiles using multiple linear regression models with the continuous TMAO-DP score as the independent variable and individual metabolites (n = 446) as the dependent variables. Crude and adjusted linear regression models were fit. Covariates were selected using former research relating dietary patterns and metabolomics profiles [30, 31, 43] and included age, smoking packyears, body mass index (BMI; kg/m^2^), recreational physical activity (MET-hrs/week), CHD case–control status, and medication use that influences atherosclerosis (hypertension medication (current/not current user), diabetes medication (current/not current user), cholesterol medication (current/not current user)). In the Discovery Sample (WHI HTs), we also adjusted for the various WHI clinical trial arms (hormone replacement therapy trial [estrogen-alone intervention, estrogen-alone control, estrogen and progestin intervention, estrogen and progestin control], dietary modification trial [not in trial, intervention, control], calcium and vitamin D trial [not in trial, intervention, control]. Bootstrap* p*-values were computed using the “boot.pval” package R with 100 iterations used to account for uncertainty in the* p*-value estimates [44–46]. The Benjamini–Hochberg method was used to calculate the false discovery rate (FDR) adjusted* p*-values to account for multiple comparisons, with an FDR adjusted *p*-value < 0.05 considered statistically significant. The metabolites found to be statistically significant in the Discovery Sample (WHI HTs) were also analyzed in the Replication Sample (WHI OS) using the same linear regression models described above. This was done to see if the same metabolites were detected in both samples.

A heatmap was created to visualize the Spearman correlations between replicated metabolites. The metabolites TMAO and choline, the TMAO-DP, and food groups were also included in the heatmap. Hierarchical clustering with complete linkage was used to group the metabolites in the heatmap.

#### Pathway enrichment analysis

Pathway enrichment analysis was performed to identify overrepresented pathways within our set of differentially expressed metabolites. KEGG IDs matched each metabolite to the metabolic pathways they participate in using the “KEGGREST” R package [47]. Mapping the entire dataset resulted in 232 of the 446 metabolites mapped to KEGG pathways, and there were 269 pathways identified. Fisher’s exact test was used to test for overrepresentation using a 2 × 2 table with mapped metabolites within the study representing the background (universe) [48]. Metabolites associated with the TMAO-DP in the Discovery Sample at an FDR adjusted *p*-value < 0.1 were included in the analysis. Since only a fraction of the metabolites in our dataset mapped to pathways, a more lenient *p*-value cutoff was used to allow more metabolites into the analysis, which results in a more informative pathway analysis.

#### Sensitivity analyses

Participants from the Replication Sample (WHI OS) were excluded from the metabolomics ancillary study if they had a history of CVD events, while this exclusion criteria was not applied to those from the Discovery Sample (WHI HTs). Therefore, in a sensitivity analysis we excluded participants in the Discovery Sample with a history of CVD events (i.e., myocardial infarction or stroke). In addition, participants in the Discovery Sample could not be using postmenopausal hormone therapy at baseline; however, the Replication Sample included participants who were using postmenopausal hormone therapy at baseline. In a second sensitivity analysis, we stratified the results of the Replication Sample by postmenopausal hormone therapy status (current/past or never users).

#### Associations between food groups and metabolites

In an exploratory analysis, we examined associations between the MPED food groups [33] that are rich sources of choline and TMAO as the independent variables and individual metabolites as the dependent variables. The MPED food groups eggs (includes eggs and egg substitutes), red meat (lean meat from beef, pork, veal, lamb, and game), and processed meats (lean meat from frankfurters, sausages, and luncheon meats) were combined to form a “red meat and eggs” group (in ounce equivalents/day), and the fish food groups (fish and other seafood low in omega-3 fatty acids and fish and other seafood high in omega-3 fatty acids) were combined to form a “total fish” group (in ounce equivalents/day). Fish were evaluated separately from red meat and eggs because fish are a direct source of TMAO, in addition to being a source of choline [7]. Also explored was whether the metabolites identified as associated with “red meat and eggs" and “total fish” were different in women with high compared to low fiber intake, defined as women above and below the median fiber intake in the sample.

Analyses were conducted using SAS (version 9.4) and RStudio (version 4.2.2). A *p*-value < 0.05 was considered statistically significant for descriptive analyses. An FDR adjusted *p*-value < 0.05 was considered statistically significant for metabolite analyses.

## Results

### Sample characteristics and dietary intake

In both the Discovery Sample and the Replication Sample, participants with higher TMAO-DP scores were more likely to be younger, have a higher BMI, have lower educational attainment, be current smokers, participate in less recreational physical activity, and take anti-diabetic medication compared to those with lower scores (Table 1). Higher TMAO-DP scores were associated with lower intake of fruits and vegetables (besides white potatoes), dairy products, fish high in omega-3 fatty acids, legumes, whole grains, and alcohol, and higher intake of red meat, processed meat, eggs, nuts and seeds, non-whole grains, and discretional fats (Table 2). In addition, higher TMAO-DP scores were associated with higher intakes of energy, percent of energy (%kcal) from protein, and %kcal fat, and lower %kcal from carbohydrates, grams of fiber, and many vitamins (Supplemental Table 2).

**Table 2:** Mean (SD) daily intakes of energy adjusted MyPyramid food groups in the Discovery Sample and the Replication Sample, by quartiles of the TMAO-DP

Food groups	Discovery sample N = 1117			Replication sample n = 870		
	Q1 [− 3.39, –0.66] n = 279	Q4 [0.70, 3.05] n = 279	*p*-value	Q1 [− 4.04, − 0.68] n = 217	Q4 [0.69, 2.68] n = 217	*p*-value
Fruit (in cup equivalents)						
Fruits (excluding citrus, melon, berries) (non-juice)	0.70 (0.52)	0.22 (0.22)	** < 0.0001**	0.79 (0.63)	0.29 (0.31)	** < 0.0001**
Citrus, melon, berries (non-juice)	0.32 (0.31)	0.12 (0.17)	** < 0.0001**	0.37 (0.33)	0.14 (0.22)	** < 0.0001**
Vegetables (in cup equivalents)						
Dark green vegetables	0.07 (0.08)	0.04 (0.05)	** < 0.0001**	0.09 (0.12)	0.06 (0.09)	**0.0020**
Orange vegetables	0.13 (0.09)	0.09 (0.07)	** < 0.0001**	0.15 (0.11)	0.10 (0.09)	** < 0.0001**
Other vegetables	0.43 (0.24)	0.32 (0.20)	** < 0.0001**	0.46 (0.30)	0.33 (0.23)	** < 0.0001**
White potato	0.15 (0.10)	0.22 (0.14)	** < 0.0001**	0.14 (0.10)	0.21 (0.16)	** < 0.0001**
Starchy vegetables	0.05 (0.06)	0.03 (0.04)	**0.0028**	0.04 (0.04)	0.04 (0.05)	0.5252
Tomato	0.21 (0.14)	0.15 (0.10)	** < 0.0001**	0.20 (0.13)	0.16 (0.13)	**0.0077**
Meat/Protein foods (in ounce equivalents unless otherwise specified)						
Franks, sausages, luncheon meats	0.21 (0.26)	0.39 (0.33)	** < 0.0001**	0.15 (0.21)	0.36 (0.33)	** < 0.0001**
Beef, pork, veal, lamb, and game	0.70 (0.49)	1.42 (0.83)	** < 0.0001**	0.52 (0.44)	1.20 (0.81)	** < 0.0001**
Chicken, turkey, and other poultry	0.53 (0.39)	0.59 (0.48)	0.3998	0.59 (0.54)	0.54 (0.44)	0.0585
Fish and other seafood high in omega-3	0.21 (0.26)	0.14 (0.21)	**0.0056**	0.28 (0.33)	0.17 (0.25)	**0.0030**
Fish and other seafood low in omega-3	0.15 (0.17)	0.19 (0.18)	0.0596	0.18 (0.19)	0.22 (0.26)	0.1021
Organ meats	0.03 (0.07)	0.03 (0.07)	0.9429	0.02 (0.05)	0.03 (0.11)	0.1507
Eggs	0.16 (0.19)	0.31 (0.33)	** < 0.0001**	0.13 (0.15)	0.31 (0.33)	** < 0.0001**
Nuts and seeds	0.16 (0.24)	0.24 (0.34)	**0.0034**	0.20 (0.28)	0.23 (0.39)	0.6604
Legumes (cup equivalents)	0.06 (0.07)	0.05 (0.07)	**0.0005**	0.07 (0.07)	0.04 (0.06)	**0.0003**
Soy product (excluding soymilk)	0.02 (0.05)	0.01 (0.05)	0.1138	0.04 (0.15)	0.02 (0.26)	0.5505
Dairy (in cup equivalents)						
Milk (including soymilk)	0.75 (0.61)	0.48 (0.45)	** < 0.0001**	0.86 (0.73)	0.47 (0.47)	** < 0.0001**
Cheese	0.28 (0.20)	0.27 (0.19)	**0.0221**	0.28 (0.22)	0.23 (0.20)	0.1529
Yogurt	0.23 (0.20)	0.01 (0.02)	** < 0.0001**	0.29 (0.28)	0.01 (0.02)	** < 0.0001**
Grains (in ounce equivalents)						
Whole grain	0.91 (0.63)	0.51 (0.45)	** < 0.0001**	0.91 (0.58)	0.61 (0.56)	** < 0.0001**
Non-whole grain	2.13 (0.75)	2.37 (0.83)	**0.0037**	2.07 (0.78)	2.42 (0.83)	** < 0.0001**
Discretionary foods						
Discretionary solid fat (grams)	15.83 (5.88)	23.46 (6.16)	** < 0.0001**	12.56 (5.39)	22.18 (6.80)	** < 0.0001**
Discretionary oil (grams)	8.19 (4.42)	12.43 (5.47)	** < 0.0001**	7.34 (4.26)	11.57 (5.28)	** < 0.0001**
Added sugars (teaspoon equivalents)	6.05 (2.47)	5.47 (3.36)	**0.0425**	6.44 (2.66)	5.73 (3.33)	**0.0114**
Alcohol (total drinks)	0.27 (0.51)	0.08 (0.31)	** < 0.0001**	0.31 (0.53)	0.12 (0.37)	** < 0.0001**

MPEDS were energy adjusted using the density method and presented as intake per 1,000 kcal. *p*-value from ANOVA

Boldface indicates statistical significance at a p-value<0.05

TMAO-DP, trimethylamine N-oxide dietary pattern; WHI, Women’s Health Initiative; Q, quartile; SD, standard deviation

### Associations between the TMAO-DP and metabolites

**Fig. 2:**
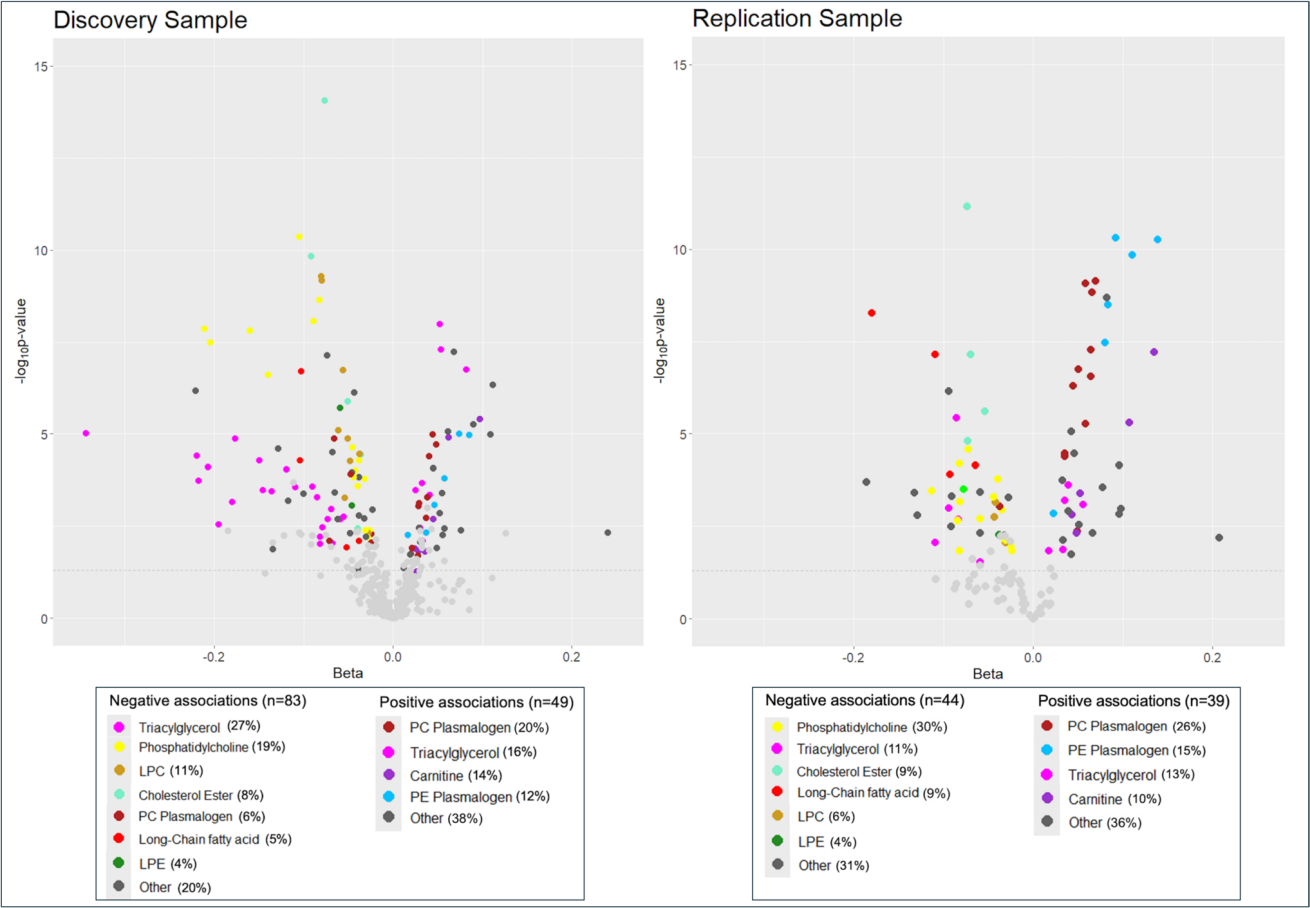
Volcano plots presenting the beta coefficients and -log_10_p-value from adjusted linear regression models evaluating associations between the TMAO-DP and metabolites in the Discovery and Replication Samples. The color of the metabolite on the plot corresponds to the metabolite class, as indicated in the figure legend below the plots. TMAO-DP, trimethylamine n-oxide dietary pattern; LPC, lysophosphatidylcholine; PC, phosphatidylcholine; LPE, lysophosphatidylethanolamine; PE, phosphatidylethanolamine

In the Discovery Sample, 157 metabolites were associated with the TMAO-DP in the unadjusted models at an FDR adjusted *p*-value < 0.05 (Supplemental Table 3). In adjusted models, 132 metabolites were significantly associated with the TMAO-DP (Supplemental Table 4; Fig. 2). Only those significant 132 metabolites were moved forward for analysis in the Replication Sample. In the unadjusted models in the Replication Sample, 91 metabolites were associated with the TMAO-DP (Supplemental Table 5), and 83 metabolites were replicated in the adjusted models (Supplemental Table 6). Of those 83 replicated metabolites, 39 metabolites had positive associations with the TMAO-DP, which were primarily phosphatidylcholine (PC) plasmalogens (n = 10; 26%), phosphatidylethanolamine (PE) plasmalogens (n = 6; 15%), triacylglycerols (TAG) (n = 5; 13%), and acyl carnitines (n = 4; 10%), and 44 metabolites had negative associations with the TMAO-DP, which were primarily PC (n = 13; 30%), TAG (5; 11%), cholesterol esters (CE) (n = 4; 9%), and long-chain fatty acids (n = 4; 9%) (Fig. 2). TMAO was statistically significantly, positively associated with the TMAO-DP in the Discovery Sample but not the Replication Sample.

**Table 3:** Overrepresented metabolic pathways among metabolites significantly associated with the TMAO-DP, identified through pathway enrichment analysis

KEGG pathway ID	Pathway	Pathway class	*p*-value	Number of metabolites mapped to pathway	Number of significant metabolites in pathway	Significant metabolites
map00564	Glycerophospholipid metabolism	Metabolism; Lipid metabolism	0.0011	86	42	C36:3 PE, C36:2 PE plasmalogen, C40:6 PC-B, C40:6 PC-A, C18:0 LPC, C18:0 LPC-B, C18:0 LPC-A, C24:0 LPC, C36:1 PC, C38:3 PC, C32:0 PC, C16:0 LPC, C34:1 PC, C40:6 PS, C40:5 PC, C22:5 CE, C40:10 PC, C40:6 PC-B, C40:6 PC-A, C38:6 PC, C36:0 PE, C38:6 PE, C15:0 LPC, C20:3 LPC, C18:3 LPC, C16:1 LPC, C22:6 LPC, alpha-glycerophosphocholine, C14:0 LPC-A, C14:0 LPC, C14:0 LPC-B, C14:0 LPC-A, C14:0 LPC-B, C14:0 LPC, C36:5 PC, C32:1 PC, C30:0 PC, C31:1 PC, C30:1 PC, C28:0 PC, C34:5 PC, C18:1 LPC
map05231	Choline metabolism in cancer	Metabolism; Lipid metabolism	0.0059	85	41	C36:4 DAG-B, C36:4 DAG-A, C36:3 DAG, C40:6 PC-A, C40:6 PC-B, C18:0 LPC, C18:0 LPC-A, C18:0 LPC-B, C36:1 PC, C24:0 LPC, C38:3 PC, C32:0 PC, C16:0 LPC, C34:1 PC, C40:5 PC, C22:5 CE, C40:10 PC, C40:6 PC-A, C40:6 PC-B, C38:6 PC, C15:0 LPC, C20:3 LPC,C18:3 LPC, C16:1 LPC, C22:6 LPC, alpha-glycerophosphocholine, C14:0 LPC, C14:0 LPC-B, C14:0 LPC-A, C14:0 LPC-A, C14:0 LPC-B, C14:0 LPC, C36:5 PC, C32:1 PC, C30:0 PC, C32:2 DAG, C31:1 PC, C30:1 PC, C28:0 PC, C34:5 PC, C18:1 LPC
map00591	Linoleic acid metabolism	Metabolism; Lipid metabolism	0.0119	41	21	C40:6 PC-A, C40:6 PC-B, C24:0 LPC, C36:1 PC, C38:3 PC, C32:0 PC, C34:1 PC, 8,11,14-eicosatrienoic acid, C40:5 PC, C40:10 PC, C40:6 PC-B, C40:6 PC-A, C38:6 PC, C36:5 PC, C32:1 PC, C30:0 PC, C31:1 PC, C30:1 PC, C28:0 PC, C34:5 PC, linoleate
map00592	alpha-Linolenic acid metabolism	Metabolism; Lipid metabolism	0.0220	37	19	C40:6 PC-A, C40:6 PC-B, C24:0 LPC, C36:1 PC, C38:3 PC, C32:0 PC, C34:1 PC, C40:5 PC, C40:10 PC, C40:6 PC-A, C40:6 PC-B, C38:6 PC, C36:5 PC, C32:1 PC, C30:0 PC, C31:1 PC, C30:1 PC, C28:0 PC, C34:5 PC
map04977	Vitamin digestion and absorption	Organismal Systems; Digestive system	0.0222	43	18	C54:6 TAG, C52:4 TAG, C54:4 TAG, C54:3 TAG, C56:5 TAG, C52:3 TAG, C18:3 CE, C22:6 CE, C20:3 CE, C48:2 TAG, C50:0 TAG, C58:8 TAG, C48:1 TAG, C16:1 CE, C20:5 CE, C52:0 TAG, thiamine, C60:12 TAG
map04148	Efferocytosis	Cellular Processes; Transport and catabolism	0.0225	50	24	C18:0 LPC, C18:0 LPC-A, C18:0 LPC-B, C18:3 CE, C16:0 LPC, C40:6 PS, C22:5 CE, C22:6 CE, C15:0 LPC, C20:3 LPC, C20:3 CE, C18:3 LPC, C16:1 LPC, C22:6 LPC, C16:1 CE, C14:0 LPC, C14:0 LPC-B, C14:0 LPC-A, C14:0 LPC, C14:0 LPC-B, C14:0 LPC-A, C20:5 CE, C18:1 LPC, putrescine
map05417	Lipid and atherosclerosis	Human Diseases; Cardiovascular disease	0.0298	41	17	C54:6 TAG, C52:4 TAG, C54:4 TAG, C54:3 TAG, C56:5 TAG, C52:3 TAG, C18:3 CE, C22:6 CE, C20:3 CE, C48:2 TAG, C50:0 TAG, C58:8 TAG, C48:1 TAG, C16:1 CE, C20:5 CE, C52:0 TAG, C60:12 TAG
map04979	Cholesterol metabolism	Organismal Systems; Digestive system	0.0387	42	17	C54:6 TAG, C52:4 TAG, C54:4 TAG, C54:3 TAG, C56:5 TAG, C52:3 TAG, C18:3 CE, C22:6 CE, C20:3 CE, C48:2 TAG, C50:0 TAG, C58:8 TAG, C48:1 TAG, C16:1 CE, C20:5 CE, C52:0 TAG, C60:12 TAG
map00590	Arachidonic acid metabolism	Metabolism; Lipid metabolism	0.0405	39	19	C40:6 PC-B, C40:6 PC-A, C24:0 LPC, C36:1 PC, C38:3 PC, C32:0 PC, C34:1 PC, C40:5 PC, C40:10 PC, C40:6 PC-B, C40:6 PC-A, C38:6 PC, C36:5 PC, C32:1 PC, C30:0 PC, C31:1 PC, C30:1 PC, C28:0 PC, C34:5 PC

Lipid metabolites are reported at the species level using sum composition annotations (i.e., number of carbon atoms:number of double bond equivalents) [38]

TMAO, trimethylamine N-oxide; DP, dietary pattern; CE, cholesterol ester; DAG, diacylglycerol; LPC, lysophosphatidylcholine; PC, phosphatidylcholine; PE, phosphatidylethanolamine; PS, phosphatidylserine; TAG, triacylglycerol

Correlations with hierarchical clustering among the metabolites validated in the Replication Sample, along with TMAO, choline, the TMAO-DP score, and food groups are presented in a heat map (Supplemental Fig. 2). Many PCs, LPCs, CEs, and TAGs are positively correlated, shown in cluster A. In cluster B, PC and PE plasmalogens correlate positively with each other (correlation coefficients ranging from 0.34–0.88). Strong positive correlations (correlation coefficients ranging from 0.71–0.93) are present among many triglycerides and diglycerides, shown in cluster C. TMAO is weakly, yet positively correlated with PCs, PEs, TAGs, and plasmalogens. In addition, TMAO clustered closely to choline, the TMAO-DP score, and the food groups discretionary solid fat, discretionary oil, and red meat and eggs. Dietary fiber and fish intake clustered closely together, but separate from TMAO.

### Pathway enrichment analysis

There were 156 metabolites associated with the TMAO-DP in the Discovery Sample at an FDR adjust* p*-value < 0.1, and 69 of these mapped to KEGG pathways. In the pathway enrichment analysis, there were nine pathways that were significantly over-represented at a* pR*-value < 0.05. Five pathways were in the lipid metabolism category: glycerophospholipid metabolism, choline metabolism in cancer, linoleic acid metabolism, alpha-linolenic acid metabolism, and arachidonic acid metabolism. The other over-represented pathways were vitamin digestion and absorption, efferocytosis, lipid and atherosclerosis, and cholesterol metabolism (Table 3**).** Notably, metabolites participate in multiple pathways, and thus there is an overlap of metabolites in the significant KEGG pathways.

### Sensitivity analyses

In a sensitivity analysis, we excluded participants from the Discovery Sample (HTs) if they had a history of CVD events (n = 153 participants had a history of CVD events, n = 964 participants did not). In the adjusted models, there were 121 metabolites significantly associated with the TMAO-DP, and the metabolites classes with positive and negative associations were similar to the primary analysis (Supplementary Table 7); however, the association between the TMAO-DP and the metabolite TMAO was no longer statistically significant after excluding those with a history of CVD events. In a different sensitivity analysis among the Replication Sample (OS), we stratified the analysis by postmenopausal hormone therapy use status. There were n = 544 participants who were past/never users and n = 326 participants who were current users. In the adjusted models, among those who were past/never users of hormone therapy there were 61 metabolites significantly related to the TMAO-DP, and among the current users there were 38 significant metabolites significantly related to the TMAO-DP (Supplementary Table 8). Twenty-five of the metabolites overlapped across strata. Again, the metabolite classes with positive and negative associations among both strata mirrored that of the primary analysis.

### Associations between food groups and metabolites

There were 124 metabolites significantly associated with the combined meat and egg food groups in the crude models within the Discovery Sample (data not shown), and in the adjusted models 107 metabolites were statistically significant (Supplemental Table 9). Of those 107 significant metabolites found in the Discovery Sample, in the Replication Sample 56 were significantly associated with red meat and egg intake in the crude models (data not shown), and 35 were significant in the adjusted models (Supplemental Table 10). Stratification by fiber did not appear to modify the associations (data not shown). The metabolites that were positively associated with meat and egg consumption are similar to what we saw with the TMAO-DP, with many of the replicated metabolites for red meat and egg intake overlapping for that of the TMAO-DP (Supplemental Table 11). Red meat and egg intake was significantly, positively associated with the TMAO metabolite in the Discovery Sample but not the Replication Sample.

When evaluating total fish food group as the exposure in the Discovery Sample, there were 44 metabolites associated with fish in the crude models (data not shown) and 41 associated with fish in the adjusted models (Supplemental Table 12). In the Replication Sample, 33 metabolites were associated with fish in the crude models (data not shown) and 32 were associated in the adjusted models (Supplementary Table 13). Stratification by fiber did not appear to modify the associations (data not shown). Some of the metabolites associated with fish intake overlap with what we saw with the TMAO-DP, however, the associations were in the opposite directions (Supplementary Table 14). Again, fish intake was significantly, positively associated with TMAO in the Discovery Sample but not the Replication Sample.

## Discussion

In this study, we evaluated associations between the atherogenic TMAO-DP and individual metabolites and pathways, offering insights into the underlying metabolic processes associated with a diet predictive of plasma TMAO and choline. The TMAO-DP was developed for the purpose of furthering the understanding the mechanism linking diet to TMAO production and ultimately atherosclerosis. We found many choline-related metabolites and pathways of lipid metabolism were associated with our atherogenic TMAO-DP, independent of traditional CVD risk factors such as smoking, physical activity, and BMI.

We developed the TMAO-DP within a sample of WHI participants with the intention of applying the dietary pattern to other WHI samples to further investigate the diet-TMAO-atherosclerosis mechanism. In this analysis of a different sample of WHI participants, the TMAO-DP scores were similar to that of the sample the diet pattern was developed in. Specifically, higher TMAO-DP scores are associated with higher intake of the food groups red meat, eggs, refined grains, discretionary fat, and white potatoes, and lower intake of fruits, dairy, and whole grains, as was expected given these are food groups that contribute to higher and lower TMAO-DP scores, respectively.

Pathway enrichment analysis can provide insight to the mechanisms that the metabolites associated with the TMAO-DP are involved in, providing context to the metabolites related to overall dietary intake. Five of the overrepresented pathways were involved in lipid metabolism, including choline metabolism. The pathways cholesterol metabolism and lipid and atherosclerosis were also overrepresented. Of note, the metabolites included in the pathway enrichment analysis were associated with the TMAO-DP independent of medication use (i.e., models adjusted for anti-hypertension, anti-diabetic, and anti-hypercholesterolemia medication use) which serve as a proxy of altered metabolic processes that may influence TMAO production in the liver. These results indicate that significant fraction of the metabolites in these metabolic pathways are associated with the TMAO-DP, indicating that the atherogenic potential of the dietary pattern may be driven by altered lipid metabolism.

About two-thirds of the metabolites associated with the TMAO-DP in the Discovery Sample were also significantly associated with the TMAO-DP in the Replication Sample. Although many of the choline-related metabolites were replicated, TMAO was not. The Replication Sample had lower overall TMAO-DP scores and a lower mean relative abundance of TMAO (data not shown), potentially limiting our ability to find a statistically significant association. The lower abundance of TMAO in the Replication Sample may have been because cases of prevalent CVD at baseline were excluded from the Replication Sample but not the Discovery Sample when designing the original CHD ancillary study. In a sensitivity analysis, cases of prevalent CVD were removed from the Discovery Sample, which resulted in the association between the TMAO-DP and TMAO becoming weaker and non-significant. In addition, production of TMAO relies not only on the intake of dietary precursors, but also on the presence of certain bacteria in the gut microbiome that can metabolize choline to produce TMA, among other factors. Dietary intake influences the gut microbiome composition [49–51]. It is possible that participants with lower TMAO-DP scores in the Replication Sample may have a lower relative abundance of the bacteria that are able to metabolize choline to produce TMA, reflected by the lower concentrations of TMAO in this sample. These results indicate that the TMAO-DP may be more useful for predicting TMAO in study samples that are at a higher risk of CVD, potentially due to higher risk samples having higher TMAO concentrations due to their dietary intake and gut microbiome composition.

Many of the metabolite classes that were positively associated with the TMAO-DP reflect the foods that contribute to higher TMAO-DP scores. For example, major food sources of plasmalogens, both phosphatidylethanolamine (PE) and phosphatidylcholine (PC), are red and processed meats [52, 53]. Plasmalogens have important biological functions, such as serving as structural lipids in cellular membranes and contributing to intracellular signaling [54, 55]. In addition, in vitro studies and studies in animal models show that plasmalogens function as antioxidants [54, 56, 57]. Because of their antioxidant properties, plasmalogens have been found to be markers of oxidative stress (i.e., higher in states of oxidative stress) [58, 59]. TMAO has been shown to increase oxidative stress, leading to endothelial dysfunction in animal models and humans [60, 61], and causing inflammation in mouse models and in vitro [13]. The positive associations between the TMAO-DP with plasmalogens in our study may reflect not only the food sources of plasmalogens (e.g., red and processed meats), but also increased synthesis of plasmalogens in response to the body’s increased oxidative stress. Previous studies have found some plasmalogens to be positively associated with coronary artery disease risk [62] and other unhealthy diet patterns [63–65].

Acyl carnitines were also positively associated with the TMAO-DP. Carnitine is a metabolite that is found in many of the same foods that are high sources of choline, such as red meat. Similar to choline, carnitine can be metabolized by certain bacteria in the gut microbiome to produce TMA, which can be oxidized by the liver to produce TMAO. Therefore, it is not surprising to see carnitines positively associated with the TMAO-DP.

Phosphatidylcholines (PCs) and lysophosphatidylcholines (LPCs) were inversely associated with the TMAO-DP. This may be contrary to what would be expected given the foods contributing to high TMAO-DP scores (red meat, processed meat, eggs) are major sources of these phospholipids. However, previous studies have found that PCs and LPCs were inversely associated with unhealthy dietary patterns [63, 64], positively associated with longevity [66], and inversely associated with CVD [67–69]. PCs have many important physiological functions, which include the transport of lipids throughout the body via their role in the structure of very-low-density lipoproteins [70], and they have anti-atherogenic properties through their role in reverse cholesterol transport (RCT) [71]. LPCs are a major phospholipid found in oxidized low-density lipoproteins (LDL), and they can be inflammatory and atherogenic [72]; however, they also have anti-atherogenic and anti-inflammatory properties such as promoting RCT [73, 74], protecting LDL from oxidation [75], and inhibiting platelet aggregation [76]. The inverse associations between these metabolites and the TMAO-DP may be reflecting their anti-atherogenic potential. Further, the finding that the metabolite TMAO was positively correlated with PC, suggests that the hypothesized atherogenic potential of the TMAO-DP, mediated through TMAO, would not be due to the potentially inflammatory properties of PC.

Cholesterol esters (CEs) were also inversely associated with the TMAO-DP. Similarly, previous studies have found CEs to be inversely associated with unhealthy diet patterns [30, 31, 63], and positively associated with healthy diet patterns [63, 77]. CEs are often reflective of a plant-based diet, and have been shown to be inversely correlated with saturated and unsaturated fat, processed meat, eggs, and potatoes [63], aligning with the foods that contribute positively to the TMAO-DP. Previous studies have noted that the relationship between CEs and CVD depends on the number of carbons and double bonds found in the CEs [78]. Specifically, highly unsaturated CEs with ≥ 5 double bonds (i.e., polyunsaturated fatty acids), such as C20:5 CE, are inversely associated with CVD [79, 80]. However, we also found that CEs with low number of double bonds, including C14:0 and C16:1 CE, were inversely associated with the TMAO-DP. The CEs C14:0 and C16:1 have been predictive of CVD events and morality [67, 81]. The identified inverse associations between various CEs and the TMAO-DP may be more reflective of the dietary sources of the CEs rather than of an underlying biological mechanism.

Triacylglycerols (TAGs) were both positively and negatively associated with the TMAO-DP. The majority of dietary lipids are TAGs and TAGs can also be endogenously produced [82]. The TAGs that were positively associated with the TMAO-DP were polyunsaturated fats, which is likely reflecting of the higher consumption of oils for those adhering highly to the TMAO-DP. The TAGs that were negatively associated with the TMAO-DP were a mix of both saturated and polyunsaturated fats, which likely reflect endogenous production given major food sources did not contribute negatively to the TMAO-DP. TAGs are a major component of cholesterol and elevated plasma TAGs have been associated with risk factors of atherosclerosis; however, they also have important physiological functions such as transporting fat and providing an energy source [83, 84]. A previous metabolomics study found that TAGs with low double-bonds (e.g., ≤ 5 double-bonds, including C52:3 TAG, positively associated with the TMAO-DP), rather than total TAGs, were associated with a higher risk of CVD [67].

The exploratory analyses demonstrated that the metabolites associated with red meat and egg intake were similar to what we saw associated with the TMAO-DP. Conversely, the metabolites associated with fish intake did not align with what we saw associated with the TMAO-DP. This is somewhat surprising given fish is a source of TMAO and a source of choline. However, different types of fish and seafood have differing TMAO content. The majority of fish consumed in our study sample was canned tuna (data not shown). A recent study demonstrated that fish with higher TMAO concentrations, such as halibut and cod, increase plasma TMAO; however, fish with lower TMAO concentrations, such as canned tuna, do not [85]. As with the TMAO-DP, the metabolite TMAO was not replicated when evaluating red meat and egg intake or fish intake as the exposures. This finding may further support the idea that there is a fundamental difference (e.g., dietary intake and gut microbiome composition) between the Discovery and Replication Samples contributing to the lack of ability to replicate TMAO. In addition, in these exploratory analyses, stratification by fiber did not appear to modify the associations between food groups and metabolomic profiles or TMAO concentrations. The amount of fiber that is needed to consume to suppress the metabolism of TMA from choline has not been quantified, however, in our sample, the mean fiber intake was relatively low. It is possible that the fiber intake in our study sample was too low to detect a modifying effect.

This study has several strengths, including the use of a validated metabolomics platform and the large sample size in comparison to many other metabolomics studies which allowed for us to use Discovery and Replication Samples, adding to the robustness of the methods. Additionally, the WHI is a well-established cohort study with robust dietary and health characteristic data. This study also has limitations. This was a cross-sectional analysis, so no conclusions about causation can be inferred. The dietary data used was self-reported and therefore likely has measurement error, although the FFQ used in the WHI results in nutrient estimates similar to that of short-term dietary assessment methods [32]. A further limitation of the dietary data is that the nutrient database used to estimate nutrient intake from the FFQ does not provide information on the intake of choline or its subtypes (i.e., phosphatidylcholine and other choline esters). Evaluating how choline subtypes are associated with the TMAO-DP and metabolites could add more nuance to the understanding of the metabolic process TMAO production from overall dietary intake. The WHI sample includes postmenopausal women, and diet and metabolism can be impacted by age, sex, and age-related hormonal changes [24], so analyses should be repeated in more diverse samples before generalizing the results to other populations. In addition, the WHI sample is a relatively homogenous population with regard to dietary intake, which may have limited our ability to detect associations with metabolites. Due to decreases in estrogen, postmenopausal women are considered to be a group at risk of choline deficiency which can have adverse health outcomes [86]. This should be considered when exploring the role of choline in CVD development among this population. The methods used for the metabolomics analyses provided information on the relative abundance of metabolites, so we do not have information on actual metabolite concentrations. In addition, we were only able to evaluate the metabolites included in Broad Institute metabolomics panels, which appears to favor lipids. This is a strength for our analysis, given the TMAO-DP is a dietary pattern high in fat, and TMAO may influence atherosclerosis through altered lipid metabolism; however, it is possible that metabolomics platforms with wider coverage of other metabolite categories may find metabolic pathways other than lipid-related pathways are important. Only about half of the metabolites mapped to KEGG pathways, limiting the ability for a comprehensive assessment of all pathways associated with the TMAO-DP. Given the observational study design, there is the possibility of residual confounding.

In conclusion, in two separate samples of postmenopausal women from the WHI, we evaluated metabolites and metabolic pathways associated with the TMAO-DP, a proposed atherogenic dietary pattern that is associated with plasma choline and TMAO. These results provide hypothesis generation into the metabolic mechanisms that may link the TMAO-DP to TMAO production and atherosclerosis. Further studies are needed to evaluate the relationship between the TMAO-DP with atherosclerosis and CVD outcomes.

## Supplementary Information

Below is the link to the electronic supplementary material.

Supplementary file1 (XLSX 116 kb)

Supplementary file2 (PDF 247 kb)

Supplementary file3 (PDF 1249 kb)

## Data Availability

Data described in the manuscript, codebook, and analytic code used in this report may be accessed in a collaborative mode as described on the Women’s Health Initiative website (www.whi.org).
