# Supplementary material for: Metabolomic profiles of an atherogenic TMAO-dietary pattern among postmenopausal women
Source: Eur J Nutr. 2025 Sep 4;64(6):271. doi: 10.1007/s00394-025-03792-w (PMC12411603; doi:10.1007/s00394-025-03792-w)
Supplement: Supplementary file 2 — Supplementary file2 (PDF 247 kb) [file 394_2025_3792_MOESM2_ESM.pdf]

Study Sample

No= Discovery Sample; Yes=Replication Sample

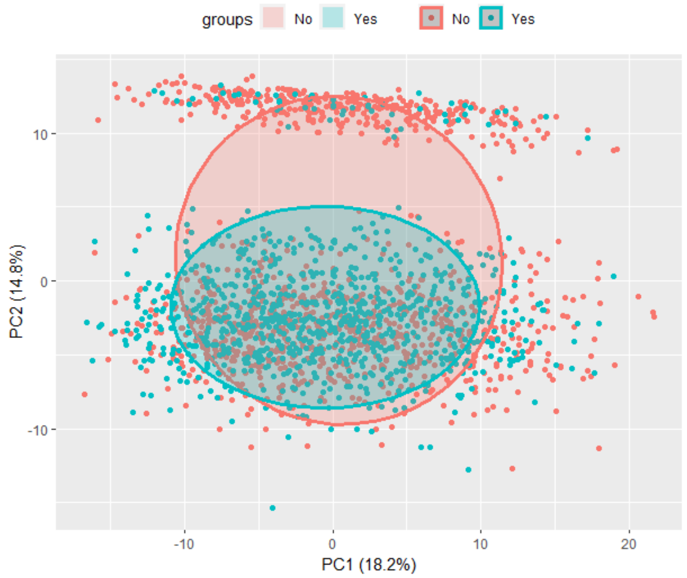

Hormone Status

0=Never/Former user; 1=Current user

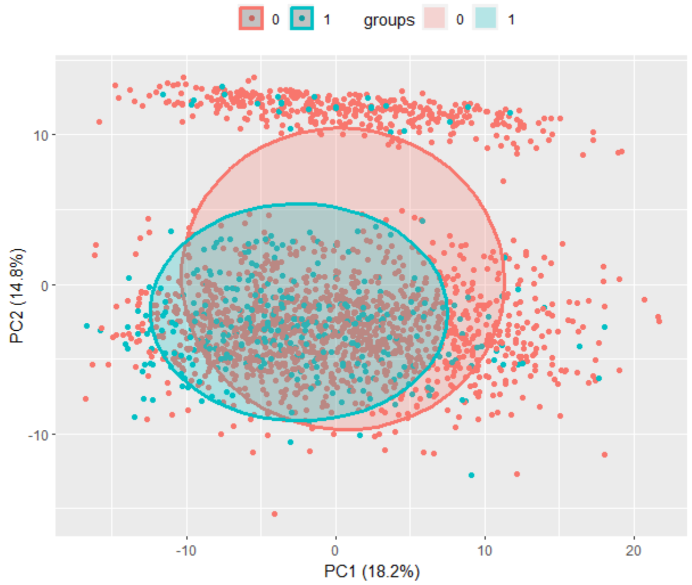

Diabetes medication

0=No; 1=Yes

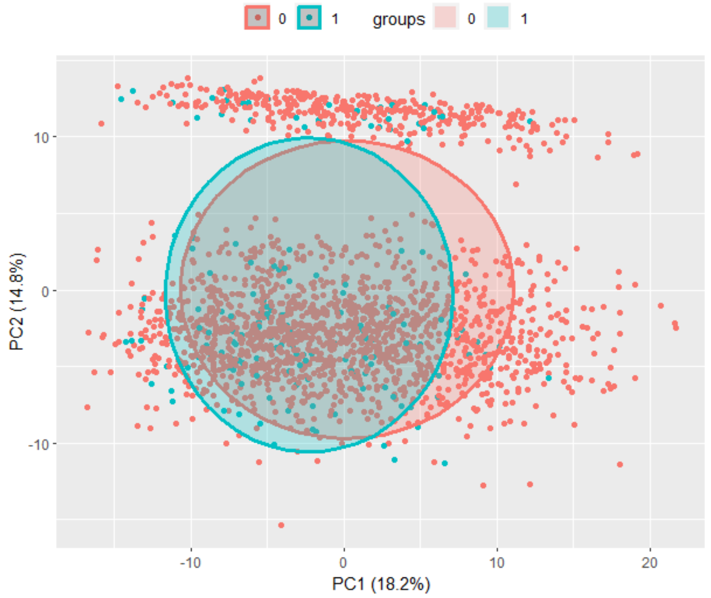

Hypertension medication

0= No; 1=Yes

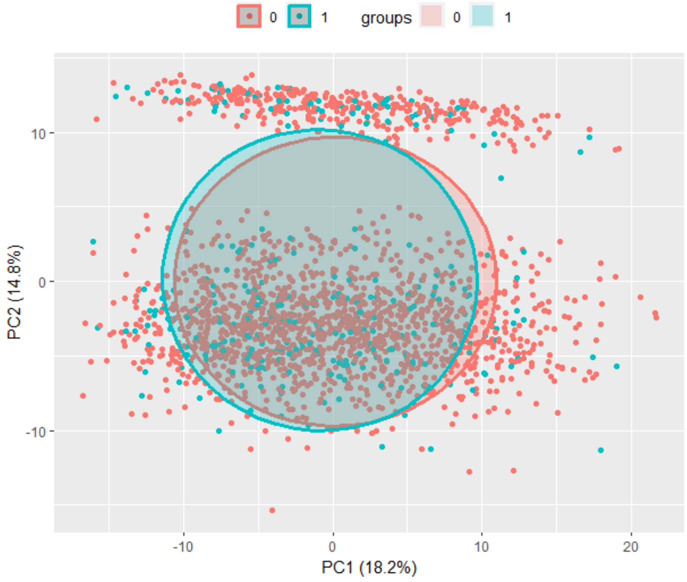

Cholesterol medication

0=No; 1=Yes

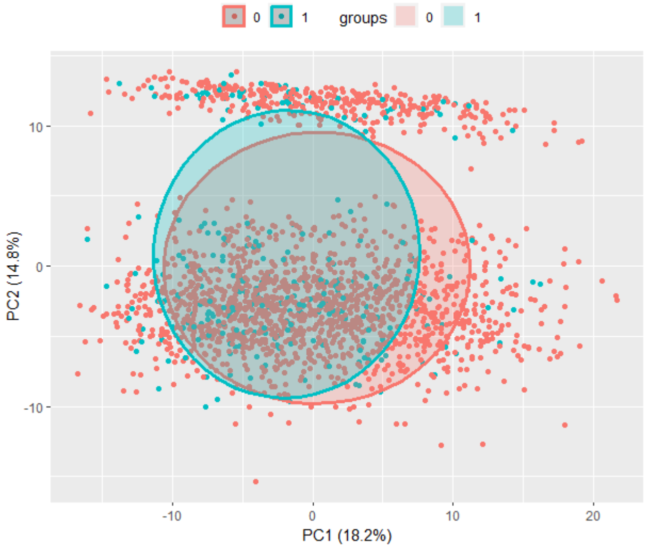

BMI Groups

1= underweight; 2= normal weight;

3= overweight; 4= obese

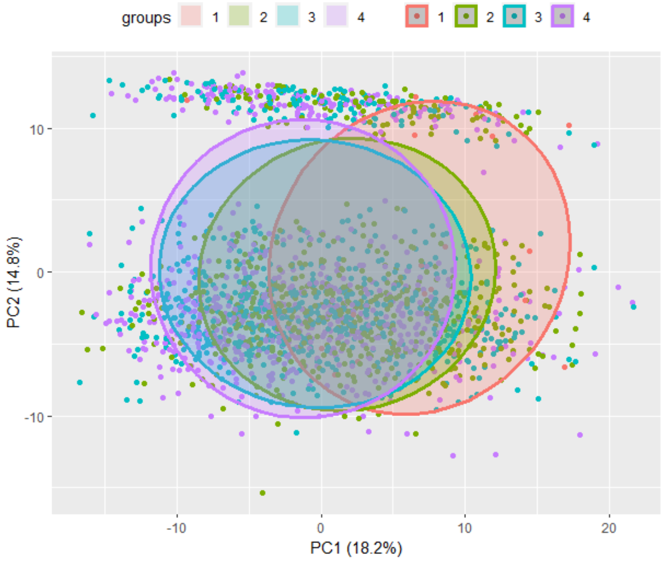

Age groups

1= <50-59; 2= 60-69; 3= 70-79+

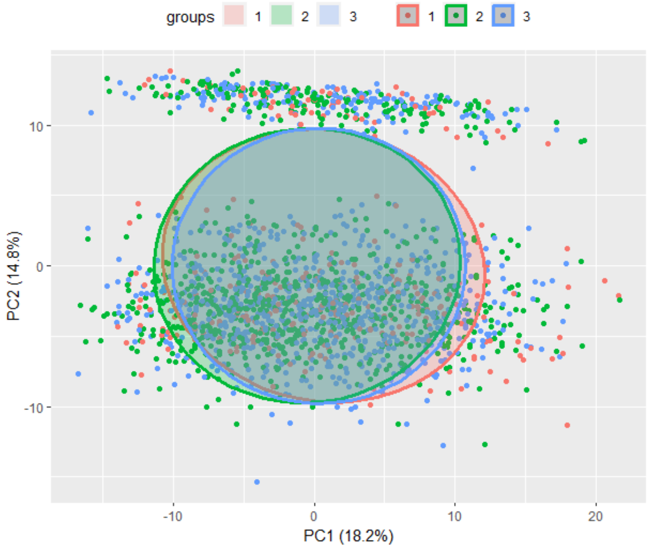

Smoking Status

1= Never; 2= Former; 3= Current

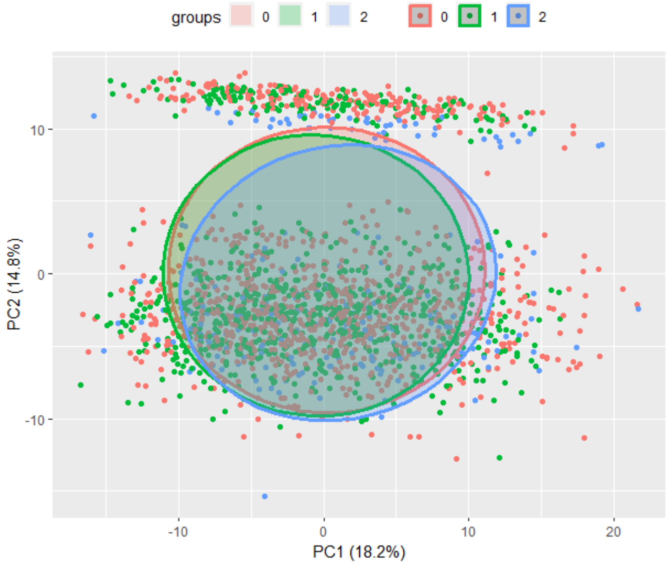

Supplemental Figure 1. Score plots plotting participants by their rank on PC1 and PC2, which explain a combined 33% variation in the metabolites. The colors on the score plots represent participant characteristics as indicated by the title and legend of each plot.
