# Supplementary material for: Metabolomic profiles of an atherogenic TMAO-dietary pattern among postmenopausal women
Source: Eur J Nutr. 2025 Sep 4;64(6):271. doi: 10.1007/s00394-025-03792-w (PMC12411603; doi:10.1007/s00394-025-03792-w)
Supplement: Supplementary file 3 — Supplementary file3 (PDF 1249 kb) [file 394_2025_3792_MOESM3_ESM.pdf]

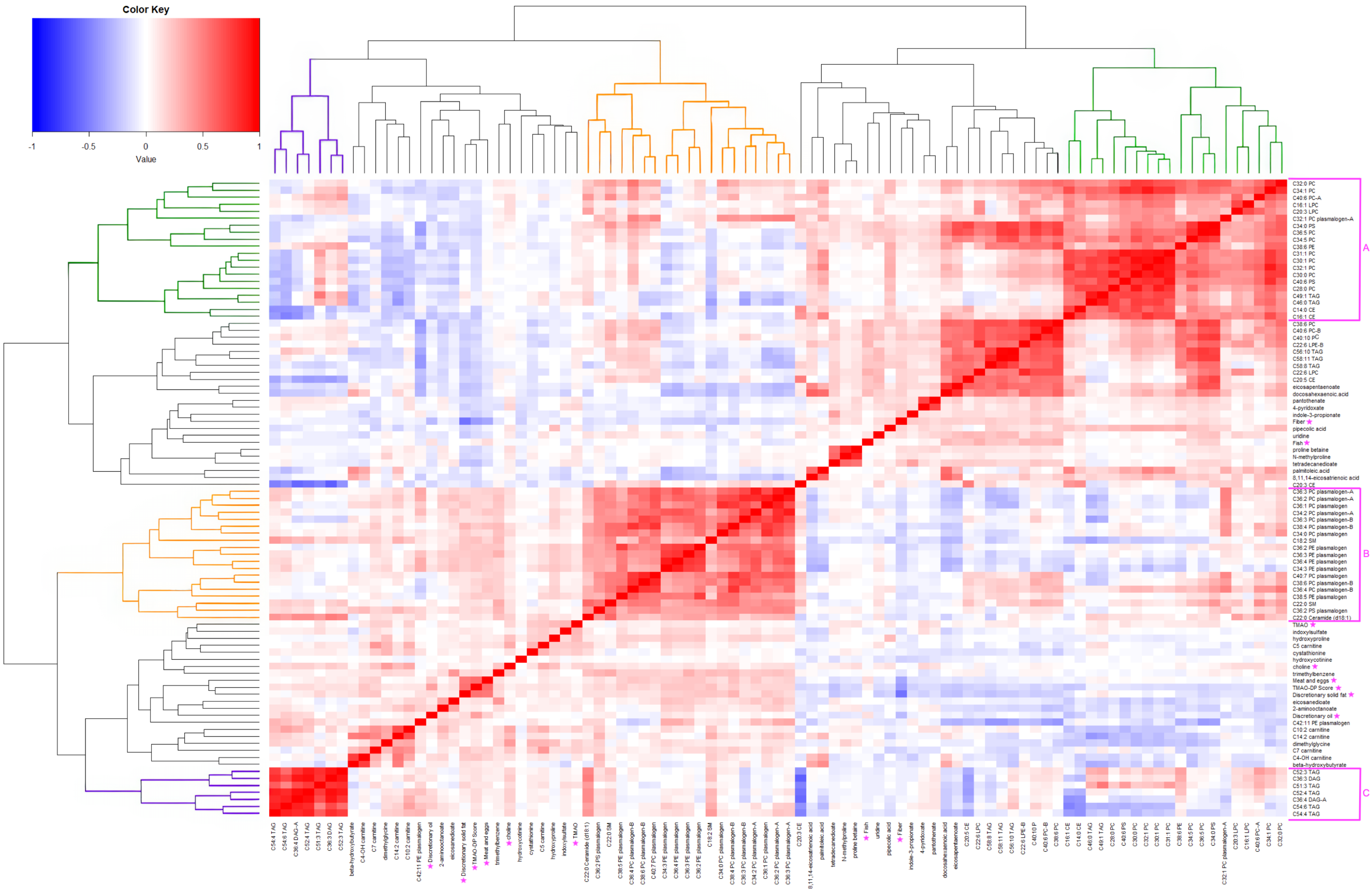
Color Key
-1
-0.5
0
0.5
1
Value
C32:0 PC
C34:1 PC
C40:6 PC-A
C16:1 LPC
C20:3 LPC
C32:1 PC plasmalogen-A
C34:0 PS
C36:5 PC
C34:5 PC
C38:6 PE
C31:1 PC
C30:1 PC
C32:1 PC
C30:0 PC
C40:6 PS
C28:0 PC
C49:1 TAG
C46:0 TAG
C14:0 CE
C16:1 CE
A
C38:6 PC
C40:6 PC-B
C40:10 PC
C22:6 LPE-B
C56:10 TAG
C58:11 TAG
C58:8 TAG
C22:6 LPC
C20:5 CE
eicosapentaenoate
docosahexaenoic.acid
pantothenate
4-pyridoxate
indole-3-propionate
Fiber ★
pipecolic acid
uridine
Fish ★
proline betaine
N-methylproline
tetradecanedioate
palmitoleic.acid
8,11,14-eicosatrienoic acid
C20:3 CE
C36:3 PC plasmalogen-A
C36:2 PC plasmalogen-A
C36:1 PC plasmalogen
C34:2 PC plasmalogen-A
C36:3 PC plasmalogen-B
C38:4 PC plasmalogen-B
C34:0 PC plasmalogen
C18:2 SM
C36:2 PE plasmalogen
C36:3 PE plasmalogen
C36:4 PE plasmalogen
C34:3 PE plasmalogen
C40:7 PC plasmalogen
C38:6 PC plasmalogen-B
C36:4 PC plasmalogen-B
C38:5 PE plasmalogen
C22:0 SM
C36:2 PS plasmalogen
C22:0 Ceramide (d18:1)
B
TMAO ★
indoxylsulfate
hydroxyproline
C5 carnitine
cystathionine
hydroxycotinine
choline ★
trimethylbenzene
Meat and eggs ★
TMAO-DP Score ★
Discretionary solid fat ★
eicosanedioate
2-aminooctanoate
Discretionary oil ★
C42:11 PE plasmalogen
C10:2 carnitine
C14:2 carnitine
dimethylglycine
C7 carnitine
C4-OH carnitine
beta-hydroxybutyrate
C52:3 TAG
C36:3 DAG
C51:3 TAG
C52:4 TAG
C36:4 DAG-A
C54:6 TAG
C54:4 TAG
C

Supplemental Figure 2. Heatmap presenting a correlation matrix of the metabolites significantly associated with the TMAO-DP in Replication Sample, plus TMAO, choline, the TMAO-DP, and food groups.

Abbreviations: CE, cholesterol ester; DAG, diacylglycerol; DP, dietary pattern; LPC, lysophosphatidylcholine; PC, phosphatidyl choline; PE, phosphatidylethanolamine; PS, phosphatidylserine; SM, sphingomyelin; TAG, triacylglycerol; TMAO, trimethylamine n-oxide

Footnotes: Metabolites, food groups, and the TMAO-DP are ordered by hierarchical clustering.
Clusters of metabolites have been labeled as clusters A, B, and C. Dendrogram branches are colored to correspond to clusters: green branches for cluster A, orange branches for cluster B, purple branches for cluster C.
TMAO, choline, the TMAO-DP, and food groups are indicated with stars.
